# Association between viral hepatitis and depressive symptoms: National Health and Nutrition Examination Survey (NHANES) 2007–2018

**DOI:** 10.1186/s12889-025-24346-z

**Published:** 2025-09-30

**Authors:** Guangli Yang, Siyuan Zhang, Yanhe Wang, Bingyu Han, Dongsheng Sun

**Affiliations:** 1 Department of General Surgery, Second Affiliated Hospital of Harbin Medical University, Harbin, Heilongjiang China; 2 Department of Neurology, Second Affiliated Hospital of Harbin Medical University, Harbin, Heilongjiang China

**Keywords:** Depressive symptoms, Viral hepatitis, HBV, HCV, NHANES, Epidemiology

## Abstract

**Background:**

Evidence on the association between viral hepatitis, particularly hepatitis B and C, and depressive symptoms remains limited. This cross-sectional study aimed to evaluate this relationship.

**Methods:**

We analyzed data from the National Health and Nutrition Examination Survey (NHANES) between 2007 and 2018. HBV or HCV infections were identified through serological testing, and depressive symptoms were measured using the Patient Health Questionnaire-9 (PHQ-9). Weighted multivariable logistic regression models were used, with additional subgroup and interaction analyses. Additionally, viral hepatitis was categorized as never infected, previously infected, or actively infected to compare risks by infection status.

**Results:**

Among 25,635 participants, 456 had HBV or HCV infection. Compared with uninfected individuals, those with viral hepatitis had higher odds of depressive symptoms (OR = 1.72, 95% CI: 1.22–2.43). When depressive symptoms were categorized by severity, viral hepatitis was associated with mild (OR = 1.46, 95% CI: 1.01–2.10), moderate (OR = 1.96, 95% CI: 1.25–3.09), and severe depression (OR = 1.74, 95% CI: 1.08–2.81). Viral hepatitis was also associated with higher PHQ-9 scores (β = 1.06, 95% CI 0.36–1.77). No significant effect modification was detected across age, sex, race/ethnicity, education level, marital status, poverty-income ratio, body mass index, smoking status or alcohol use (all *p* for interaction > 0.05). When infection status was further stratified, only active infection was significantly associated with depressive symptoms (OR = 1.67, 95% CI: 1.19–2.33).

**Conclusions:**

Viral hepatitis was independently associated with depressive symptoms, and this relationship was driven primarily by active infection. As causality cannot be inferred from cross-sectional data, longitudinal studies are warranted.

**Supplementary Information:**

The online version contains supplementary material available at 10.1186/s12889-025-24346-z.

## Introduction

Depression is one of the most prevalent mental health disorders worldwide, characterized by persistent low mood, anhedonia, and various cognitive and physiological disturbances [1]. According to World Health Organization (WHO) estimates, depression affects over 280 million individuals globally, representing approximately 3.8% of the world’s population, and ranks as a leading cause of disability among mental disorders [2]. In the United States, approximately 18.5% of adults aged 18 years or older reported a diagnosis of depression in 2020 [3], with prevalence increasing significantly during the COVID-19 pandemic [4]. Depression impairs social functioning and worsens the progression and prognosis of coexisting chronic diseases [5–7]. Thus, early identification and management of depression in individuals with chronic illnesses are essential.

Viral hepatitis, particularly chronic hepatitis B (HBV) and hepatitis C (HCV), is a major cause of liver fibrosis, cirrhosis, and hepatocellular carcinoma, posing significant global public health challenges [8]. As of 2022, WHO estimates that approximately 254 million individuals are chronically infected with HBV and around 50 million with HCV, imposing significant burdens on affected individuals, families, and healthcare systems [9]. Beyond its direct physiological effects, viral hepatitis also significantly impacts mental health, particularly by increasing susceptibility to depressive symptoms [10–12]. However, large-scale epidemiological studies on the relationship between viral hepatitis and depressive symptoms remain limited.

Therefore, this study aimed to examine the relationship between viral hepatitis and depressive symptoms using nationally representative data from the National Health and Nutrition Examination Survey (NHANES).

## Methods

### Study design and participants

NHANES is an ongoing, nationally representative, cross-sectional survey conducted by the U.S. Centers for Disease Control and Prevention (CDC). Using a complex, multistage sampling design, NHANES has collected nationwide data periodically since 1999 through household interviews, physical examinations, and laboratory tests, covering various aspects of health, nutrition, and lifestyle. The NHANES study protocol was approved by the Institutional Review Board (IRB) of the National Center for Health Statistics (NCHS), and all participants provided informed consent. Comprehensive ethical guidelines and all relevant data are publicly available on the official NHANES website (https://www.cdc.gov/nchs/nhanes/index.htm).

**Fig. 1:**
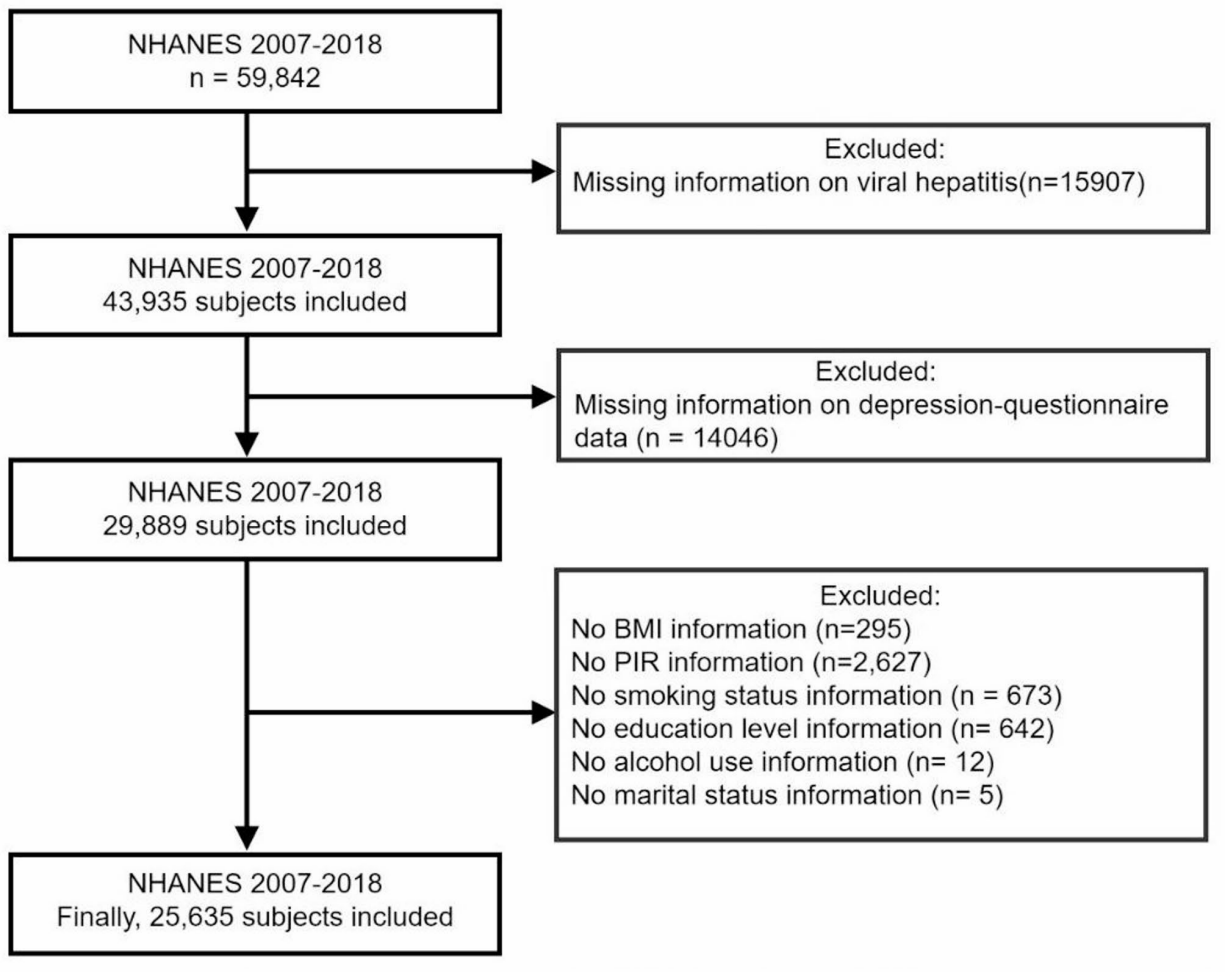
Flow chart of study selection

All analyses in this study followed NHANES guidelines and regulations. Data were obtained from NHANES surveys conducted between 2007 and 2018. The initial sample included 59,842 participants. Exclusion criteria were as follows: (1) Participants without available viral hepatitis serological data (2) Participants with incomplete depression-questionnaire (3) Participants with missing covariate data, including body mass index (BMI), poverty-income ratio (PIR), smoking status, education level, alcohol use, marital status. After these exclusions, all remaining covariates used in the analysis had no missing data. Ultimately, 25,635 participants were included in this study. A detailed flowchart of participant inclusion and exclusion is presented in Fig. 1.

### Definition of depressive symptoms

Depressive symptoms were assessed using the Patient Health Questionnaire-9 (PHQ-9), a widely used screening tool for depressive symptoms [13]. The PHQ-9 evaluates nine common depressive symptoms experienced over the past two weeks. Each item is scored on a four-point scale (0–3): “not at all”, “several days”, “more than half the days”, or “nearly every day”. Depression severity is categorized based on the total PHQ-9 score: no depression (0–4), mild [5–9], moderate [10–14], and severe (≥ 15) [14]. Furthermore, participants with a PHQ-9 score > 10 were classified as having depressive symptoms [15].

### Definition of hepatitis virus infection

Viral hepatitis infection was defined according to NHANES protocols. For hepatitis B virus (HBV), whole blood specimens were initially screened for hepatitis B core antibody (anti-HBc); only reactive samples underwent confirmatory testing for hepatitis B surface antigen (HBsAg) using the VITROS ECi/3600 immunoassay system [16]. By NHANES convention, anti-HBc–negative specimens were not retested and were considered HBsAg negative. All serum samples were screened for hepatitis C virus (HCV) antibodies (anti-HCV); reactive samples underwent quantitative HCV RNA testing using the COBAS Amplicor HCV Test v2.0 [17]. A non-reactive anti-HCV screen was therefore considered HCV RNA negative. For the primary analyses, viral hepatitis infection was defined as either HBsAg positivity or detectable HCV RNA. To explore whether past exposure differed from current infection in relation to depressive symptoms, we categorized participants’ viral hepatitis status into three groups: (1) never infected, defined as negative for HBsAg, antiHBc, and antiHCV antibodies; (2) previously infected, defined as HBsAgnegative with antiHBc positivity and/or antiHCV positivity but undetectable HCV RNA; and (3) actively infected, defined as HBsAgpositive and/or detectable HCV RNA [18, 19]. Hepatitis A virus (HAV) infection was not included in the definition, as HAV typically represents acute infection rather than chronic infection.

### Covariates

Based on a comprehensive review of the literature [14, 20] and clinical experience, this study identified several potential confounders that may affect the relationship between depressive symptoms and viral hepatitis. These variables include age (in years), sex (male/female), race/ethnicity, education level, marital status, PIR, BMI, smoking status, alcohol use, diabetes, and hypertension. Illicit drug-use variables were excluded because NHANES publicly releases these data only for participants aged 18–69 years, and the corresponding items exhibit high non-response rates.

Covariates were classified according to NHANES database standards. Race/ethnicity was divided into four groups: Mexican American, non-Hispanic Black, non-Hispanic White, and other racial/ethnic groups. Education level was categorized as below high school, high school, and above high school. Marital status was classified as “married/living with a partner” and “living alone,” with the latter including individuals who were never married, widowed, divorced, or separated. PIR was classified as low income (PIR < 1.35), middle income (1.35 ≤ PIR < 3.0), and high income (PIR ≥ 3.0). BMI was grouped into four categories: underweight (BMI < 18.5), normal weight (18.5 ≤ BMI < 25), overweight (25 ≤ BMI < 30), and obese (BMI ≥ 30). Smoking status was categorized as never smoked (fewer than 100 cigarettes in a lifetime), former smoker (more than 100 cigarettes but no longer smoking), and current smoker (more than 100 cigarettes and currently smoking, either occasionally or regularly). Alcohol use was categorized as “yes” (≥ 12 drinks in a lifetime) or “no” (< 12 drinks in a lifetime). Hypertension was defined as an average systolic blood pressure ≥ 140 mmHg and/or diastolic blood pressure ≥ 90 mmHg, a self-reported diagnosis, or use of antihypertensive medications. Diabetes was defined as a physician’s diagnosis, hemoglobin A1c (HbA1c) ≥ 6.5%, fasting blood glucose ≥ 7.0 mmol/L, random or 2-hour oral glucose tolerance test (OGTT) blood glucose ≥ 11.1 mmol/L, or use of diabetes medications or insulin.

### Statistical analysis

This study adhered to NHANES analysis and reporting guidelines, fully accounting for the complex sampling design and survey weights. Cycle-specific MEC examination weights (WTMEC2YR) were divided by six to create 2007–2018 weights, ensuring national representativeness. For descriptive analyses, categorical variables were expressed as weighted percentages, while non-normally distributed continuous variables were reported as medians with interquartile ranges (IQRs). Skewed continuous variables were analyzed using Kruskal–Wallis tests, while categorical variables were assessed using chi-square tests. Weighted logistic regression models were used to assess the relationship between viral hepatitis and depressive symptoms. Three multivariable models were constructed: Model 1 was unadjusted; Model 2 adjusted for age, sex, race/ethnicity, education level, marital status, PIR, and BMI; and Model 3 further adjusted for smoking status, alcohol use, diabetes, and hypertension. Model fit was assessed using the Akaike Information Criterion (AIC), the Bayesian Information Criterion (BIC), and the survey-weighted Wald F statistic. All models demonstrated relatively good global fit (Supplementary Table S1 in Additional file 1). In addition, stratified and interaction analyses were conducted by age (< 60 vs. ≥60 years), sex, race/ethnicity, education level, marital status, PIR, BMI, smoking status, and alcohol use to examine potential variations in the association between viral hepatitis and depressive symptoms. Results from the weighted logistic regression and subgroup analyses are reported as odds ratios (ORs) with 95% confidence intervals (CIs). All statistical analyses were carried out using RStudio (version 4.3.3), with statistical significance defined as *P* < 0.05.

## Results

### Baseline characteristics based on viral hepatitis

Table 1 presents the baseline characteristics of the study participants. Among the 25,635 participants, 456 had viral hepatitis, while 25,179 did not. Because only 1.8% of participants had viral hepatitis, a post hoc power analysis was conducted, which indicated sufficient power to detect the observed associations (Supplementary Material in Additional file 2). Compared to individuals without viral hepatitis, those with viral hepatitis were more likely to be older, male, non-Hispanic Black, have lower educational attainment and income, and were more likely to live alone. They also had a higher prevalence of current smoking and hypertension, and were less likely to be obese. No significant difference was observed in diabetes prevalence. Notably, participants with viral hepatitis had higher PHQ-9 scores (*p* < 0.001), and the prevalence of depressive symptoms was more than twice as high compared to uninfected individuals (18.4% vs. 7.8%).

**Table 1:** Characteristics of the participants in the NHANES (2007–2018)

Characteristic	Overall (*N* = 25,635)	Hepatitis Virus Infection (*N* = 456)	No Hepatitis Virus Infection (*N* = 25,179)	*P*-value
**Age (years)**	49.0 (33.0, 60.0)	54.5 (44.0, 59.0)	49.0 (33.0, 60.0)	< 0.001
**Sex**				< 0.001
Female	13,022 (51.2%)	165 (33.0%)	12,857 (51.5%)	
Male	12,613 (48.8%)	291 (67.0%)	12,322 (48.5%)	
**Race/Ethnicity**				< 0.001
Mexican American	3,761 (8.1%)	33 (4.8%)	3,728 (8.2%)	
Non-Hispanic White	11,212 (68.9%)	138 (55.6%)	11,074 (69.0%)	
Non-Hispanic Black	5,208 (10.2%)	169 (21.3%)	5,039 (10.1%)	
Other	5,454 (12.8%)	116 (18.3%)	5,338 (12.7%)	
**Education Level**				< 0.001
Below high school	5,841 (14.6%)	162 (34.0%)	5,679 (14.4%)	
High school	5,896 (23.0%)	125 (30.3%)	5,771 (22.9%)	
Above high school	13,898 (62.4%)	169 (35.7%)	13,729 (62.7%)	
**Marital Status**				< 0.001
Married/living with partner	15,318 (63.8%)	228 (50.6%)	15,090 (64.0%)	
Living alone	10,317 (36.2%)	228 (49.4%)	10,089 (36.0%)	
**PIR**				< 0.001
Low income	8,080 (21.2%)	236 (43.4%)	7,844 (20.9%)	
Middle income	9,698 (35.4%)	153 (37.2%)	9,545 (35.4%)	
High income	7,857 (43.4%)	67 (19.5%)	7,790 (43.7%)	
**BMI (kg/m²)**				< 0.001
Underweight	368 (1.4%)	7 (1.1%)	361 (1.4%)	
Normal	6,817 (27.4%)	164 (40.0%)	6,653 (27.2%)	
Overweight	8,430 (33.0%)	159 (32.4%)	8,271 (33.0%)	
Obese	10,020 (38.2%)	126 (26.5%)	9,894 (38.4%)	
**Smoking Status**				< 0.001
Never smoked	14,088 (55.3%)	130 (23.4%)	13,958 (55.7%)	
Former smoker	6,306 (25.2%)	109 (23.7%)	6,197 (25.2%)	
Current smoker	5,241 (19.5%)	217 (52.8%)	5,024 (19.1%)	
**Alcohol Use**				0.001
Yes	19,312 (80.6%)	373 (85.8%)	18,939 (80.6%)	
No	6,323 (19.4%)	83 (14.2%)	6,240 (19.4%)	
**Diabetes**				0.281
Yes	4,650 (13.6%)	92 (16.3%)	4,558 (13.5%)	
No	20,985 (86.4%)	364 (83.7%)	20,621 (86.5%)	
**Hypertension**				< 0.001
Yes	9,337 (32.2%)	211 (46.5%)	9,126 (32.0%)	
No	16,298 (67.8%)	245 (53.5%)	16,053 (68.0%)	
**PHQ-9 Score**	2.0 (0.0, 4.0)	3.0 (0.0, 8.0)	2.0 (0.0, 4.0)	< 0.001
**Depressive Symptoms**				< 0.001
No depression	19,189 (76.6%)	290 (59.6%)	18,899 (76.8%)	
Mild depression	4,121 (15.4%)	85 (22.3%)	4,036 (15.3%)	
Moderate depression	1,460 (5.1%)	44 (11.2%)	1,416 (5.0%)	
Severe depression	865 (2.9%)	37 (7.0%)	828 (2.9%)	
**Depressive Symptoms**				< 0.001
Yes	2,325 (8.0%)	81 (18.1%)	2,244 (7.9%)	
No	23,310 (92.0%)	375 (81.9%)	22,935 (92.1%)	

Continuous variables are presented as Median (Q1, Q3), categorical variables as n (%)

*Abbreviations*: *NHANES* National Health and Nutrition Examination Survey, *PIR* Poverty Income Ratio, *BMI* Body Mass Index, *PHQ-9* 9-item Patient Health Questionnaire

### Association between viral hepatitis and depressive symptoms

Table 2 shows the results of the univariate logistic regression analysis, indicating significant associations (*p* < 0.05) between depressive symptoms and multiple variables, including sex, race/ethnicity, education level, marital status, PIR, BMI, smoking status, diabetes, hypertension, and hepatitis virus infection.

**Table 2:** Associations between variables and depressive symptoms

Variable	OR (95% CI)	*P*-value
Age (years)	1.00 (1.00, 1.01)	0.146
Sex: Female vs. Male	1.84 (1.63, 2.07)	< 0.001
Race/Ethnicity: ref. = Mexican American		
Non-Hispanic White	0.97 (0.81, 1.17)	0.781
Non-Hispanic Black	0.74 (0.62, 0.89)	0.001
Other	0.78 (0.64, 0.96)	0.018
Education Level: ref. = Below high school		
High school	1.50 (1.30, 1.74)	< 0.001
Above high school	2.28 (1.96, 2.66)	< 0.001
Marital Status: ref. = Married or living with partner		
Living alone	0.49 (0.44, 0.55)	< 0.001
PIR: ref. = Low income		
Middle income	2.06 (1.81, 2.34)	< 0.001
High income	4.19 (3.55, 4.95)	< 0.001
BMI: ref. = Underweight		
Normal BMI	1.82 (1.15, 2.88)	0.011
Overweight	2.03 (1.28, 3.22)	0.003
Obese	1.20 (0.77, 1.86)	0.417
Smoking Status: ref. = Never smoked		
Former smoker	0.74 (0.63, 0.88)	< 0.001
Current smoker	0.31 (0.27, 0.35)	< 0.001
Alcohol Use: Yes vs. No	1.00 (0.89, 1.13)	0.968
Diabetes: Yes vs. No	1.62 (1.41, 1.86)	< 0.001
Hypertension: Yes vs. No	1.68 (1.49, 1.90)	< 0.001
Hepatitis Virus Infection: Yes vs. No	2.60 (1.88, 3.58)	< 0.001

Results are based on weighted data

*OR* odds ratio, *CI* confidence interval, *Ref* reference

Table 3 presents the results of the multivariate logistic regression analyses examining the relationship between viral hepatitis and depressive symptoms (PHQ-9 ≥ 10). In the unadjusted model (Model I), participants with viral hepatitis had significantly higher odds of depressive symptoms compared to those without (OR = 2.60, 95% CI: 1.88–3.58; *p* < 0.001). This association remained robust after adjusting for age, sex, race/ethnicity, education level, marital status, PIR, and BMI in Model II (OR = 2.13, 95% CI: 1.53–2.96; *p* < 0.001). In the fully adjusted Model III, which additionally controlled for smoking status, alcohol use, diabetes, and hypertension, the association persisted (OR = 1.66, 95% CI: 1.18–2.33; *p* = 0.004).

**Table 3:** Logistic regression analyses for association between viral hepatitis and depressive symptoms (PHQ-9 ≥ 10)

Variable	Depressive symptoms		*P*-value
	No (Ref)	Yes (OR [95% CI])	
Model I	1 (Ref)	2.60 (1.88, 3.58)	< 0.001
Model II	1 (Ref)	2.13 (1.53, 2.96)	< 0.001
Model III	1 (Ref)	1.66 (1.18, 2.33)	0.004

This table presents the results from a multivariable regression analysis, adjusted for potential confounders. All analyses have been weighted to account for the survey’s complex sampling design

*CI* Confidence Interval, *OR* Odds Ratio, *Ref* Reference

Model I: Unadjusted

Model II: Adjusted for age, sex, race/ethnicity, education level, marital status, PIR, BMI

Model III: Adjusted for age, sex, race/ethnicity, education level, marital status, PIR, BMI, smoking status, alcohol use, diabetes, hypertension

Table 4 presents the logistic regression results for the association between viral hepatitis and depressive symptom severity, categorized as mild, moderate, and severe based on PHQ-9 scores. In the unadjusted model (Model I), participants with viral hepatitis had significantly higher odds of mild (OR = 1.87, 95% CI: 1.31–2.69; *p* < 0.001), moderate (OR = 2.88, 95% CI: 1.92–4.32; *p* < 0.001), and severe (OR = 3.14, 95% CI: 1.96–5.03; *p* < 0.001) depressive symptoms compared to those without viral hepatitis. After adjusting for age, sex, race/ethnicity, education level, marital status, PIR, and BMI in Model II, the associations remained statistically significant for mild (OR = 1.76, 95% CI: 1.23–2.50; *p* = 0.002), moderate (OR = 2.51, 95% CI: 1.63–3.87; *p* < 0.001), and severe (OR = 2.36, 95% CI: 1.50–3.72; *p* < 0.001) depressive symptoms. In the fully adjusted Model III, which additionally controlled for smoking status, alcohol use, diabetes, and hypertension, viral hepatitis remained significantly associated with increased odds of mild (OR = 1.50, 95% CI: 1.04–2.17; *p* = 0.031), moderate (OR = 1.89, 95% CI: 1.21–2.95; *p* = 0.006), and severe (OR = 1.68, 95% CI: 1.05–2.68; *p* = 0.032) depressive symptoms.

**Table 4:** Logistic regression analyses for association between viral hepatitis and PHQ-9 severity categories

Depressive symptoms	Model I OR (95% CI)	*P*-value	Model II OR (95% CI)	*P*-value	Model III OR (95% CI)	*P*-value
No	1 (Ref)	-	1 (Ref)	-	1 (Ref)	-
Mild	1.87 (1.31, 2.69)	< 0.001	1.76 (1.23, 2.50)	0.002	1.50 (1.04, 2.17)	0.031
Moderate	2.88 (1.92, 4.32)	< 0.001	2.51 (1.63, 3.87)	< 0.001	1.89 (1.21, 2.95)	0.006
Severe	3.14 (1.96, 5.03)	< 0.001	2.36 (1.50, 3.72)	< 0.001	1.68 (1.05, 2.68)	0.032

This table presents the results from a multivariable regression analysis, adjusted for potential confounders. All analyses have been weighted to account for the survey’s complex sampling design

*CI* Confidence Interval, *OR* Odds Ratio, *Ref* Reference

Model I: Unadjusted

Model II: Adjusted for age, sex, race/ethnicity, education level, marital status, PIR, BMI

Model III: Adjusted for age, sex, race/ethnicity, education level, marital status, PIR, BMI, smoking status, alcohol use, diabetes, hypertension

Table 5 presents the linear-regression results for the association between viral hepatitis and PHQ-9 score. Viral hepatitis was associated with higher PHQ-9 scores in all models: Model I (β = 1.86, 95% CI: 1.10–2.63; *p* < 0.001), Model II (β = 1.53, 95% CI: 0.84–2.22; *p* < 0.001), Model III (β = 1.06, 95% CI: 0.36–1.77; *p* = 0.004).

**Table 5:** Linear-regression analyses for association between viral hepatitis and PHQ-9 score

Variable	PHQ-9 score	*P*-value
	β (95%CI)	
Model I	1.86 (1.10, 2.63)	< 0.001
Model II	1.53 (0.84, 2.22)	< 0.001
Model III	1.06 (0.36, 1.77)	0.004

This table presents the results from a multivariable regression analysis, adjusted for potential confounders. All analyses have been weighted to account for the survey’s complex sampling design

*CI* Confidence Interval

Model I: Unadjusted

Model II: Adjusted for age, sex, race/ethnicity, education level, marital status, PIR, BMI

Model III: Adjusted for age, sex, race/ethnicity, education level, marital status, PIR, BMI, smoking status, alcohol use, diabetes, hypertension

Table 6 presents the association between viral hepatitis status and depressive symptoms. In the unadjusted model (Model I), participants with previous infection had higher odds of depressive symptoms than those never infected (OR = 1.25, 95% CI 1.01–1.56; *p* = 0.043), and the association was even stronger among those with active infection (OR = 2.63, 95% CI 1.90–3.62; *p* < 0.001). After adjustment for age, sex, race/ethnicity, education level, marital status, PIR, BMI (Model II), the association for past infection was attenuated and became nonsignificant (OR = 1.15, 95% CI 0.91–1.44), whereas active infection remained robust (OR = 2.15, 95% CI 1.55–2.99; *p* < 0.001). Further adjustment for smoking status, alcohol use, diabetes, and hypertension (Model III) yielded an OR of 1.67 (95% CI 1.19–2.33; *p* = 0.003) for active infection, while the estimate for past infection remained nonsignificant (OR = 1.08, 95% CI 0.86–1.36; *p* = 0.511). Thus, only active viral hepatitis was independently associated with depressive symptoms.

**Table 6:** Logistic regression analyses for association between viral hepatitis status and depressive symptoms

Viral Hepatitis Status	Model I OR (95% CI)	*P*-value	Model II OR (95% CI)	*P*-value	Model III OR (95% CI)	*P*-value
Never infected	1 (Ref)	-	1 (Ref)	-	1 (Ref)	-
Previous infection	1.25 (1.01,1.56)	0.043	1.15 (0.91,1.44)	0.234	1.08 (0.86,1.36)	0.511
Active infection	2.63 (1.90,3.62)	< 0.001	2.15 (1.55,2.99)	< 0.001	1.67 (1.19,2.33)	0.003

This table presents the results from a multivariable regression analysis, adjusted for potential confounders. All analyses have been weighted to account for the survey’s complex sampling design

*CI* Confidence Interval, *OR* Odds Ratio, *Ref* Reference

Model I: Unadjusted

Model II: Adjusted for age, sex, race/ethnicity, education level, marital status, PIR, BMI

Model III: Adjusted for age, sex, race/ethnicity, education level, marital status, PIR, BMI, smoking status, alcohol use, diabetes, hypertension

### Subgroup analyses for the associations

**Fig. 2:**
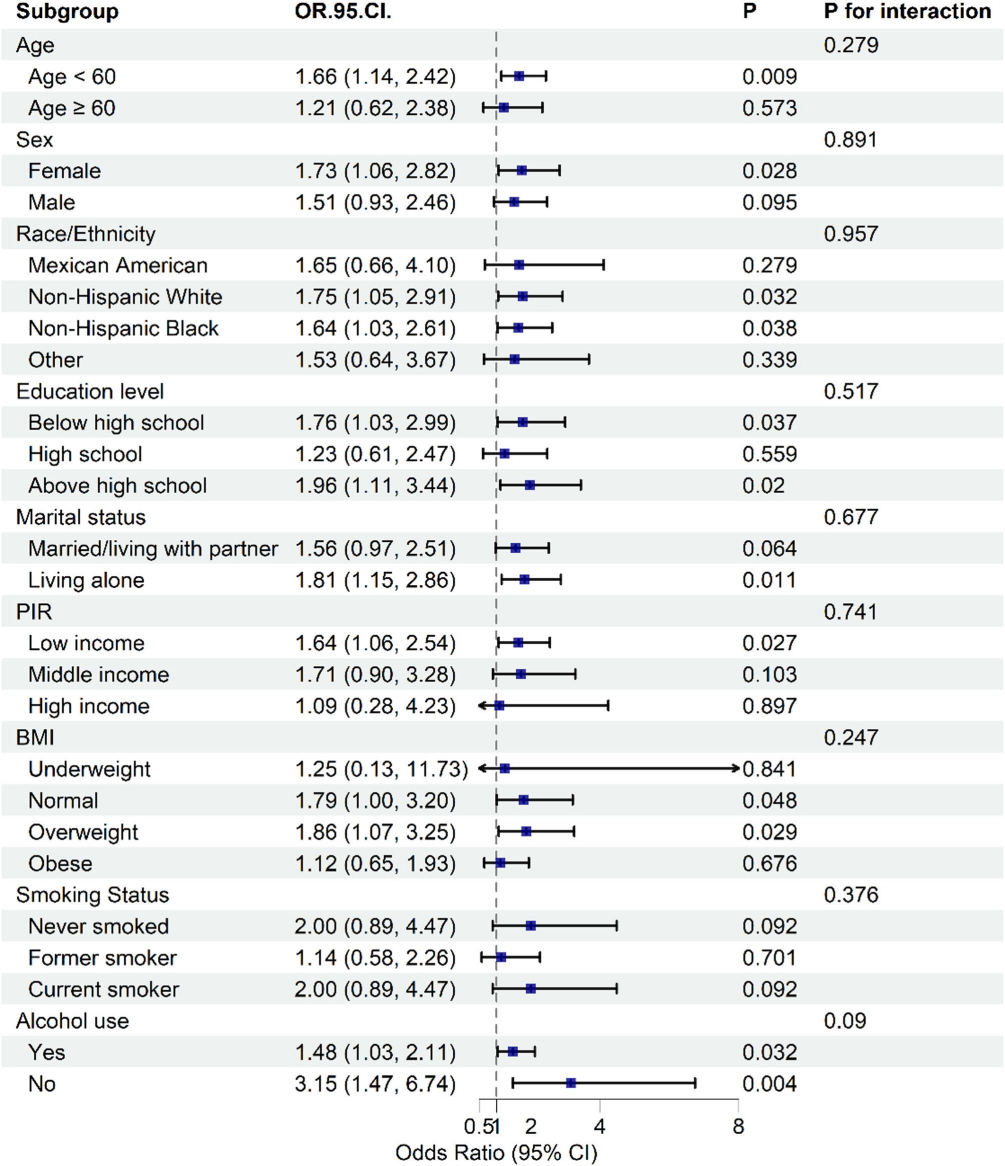
Forest plot of subgroup analyses showing the association between viral hepatitis and depressive symptoms, stratified by age, sex, race/ethnicity, education level, marital status, PIR, BMI, smoking status, and alcohol use

To assess the consistency of the association between viral hepatitis and depressive symptoms across different population subgroups, we conducted stratified and interaction analyses. Figure 2 presents the forest plot of the subgroup analyses. No statistically significant differences were detected in the association between viral hepatitis and depressive symptoms across different subgroups, indicating that age, sex, race/ethnicity, education level, marital status, PIR, BMI, smoking status, and alcohol use did not significantly affect this positive correlation (all p for interaction > 0.05).

## Discussion

In this nationally representative cross-sectional study of 25,635 NHANES participants, viral hepatitis was significantly associated with depressive symptoms. This association remained statistically significant across all multivariable models after adjustment for relevant covariates, and remained robust across subgroups defined by age, sex, race/ethnicity, education level, marital status, PIR, BMI, smoking status, and alcohol use. Moreover, when viral hepatitis was further categorized into never infected, previously infected, and actively infected, only active infection remained independently associated with depressive symptoms after full adjustment. This finding suggests that ongoing viral activity —rather than prior exposure—may be a key factor contributing to psychological distress. These findings support routine mental health screening in the management of viral hepatitis.

Our findings are consistent with earlier evidence linking viral hepatitis to depressive outcomes. Previous research has detected viral genetic material in the central nervous system. Radkowski et al. and Laskus et al. detected HCV RNA sequences in brain tissue and cerebrospinal fluid (CSF) [21, 22]. Similarly, Ene et al. (2015) provided the first evidence of HBV DNA in the CSF of HIV-infected patients [23]. The direct presence of viral genetic material in the central nervous system suggests that chronic hepatitis infections might directly impair brain function and potentially trigger the development of depression [24]​. Beyond these mechanistic insights, epidemiological data also support a robust association between viral hepatitis and depression. Yates and Gleason (1998) were among the first to report that individuals with chronic HCV infection exhibit a higher prevalence of depression than the general population [25]. This observation has been corroborated by multiple studies, with estimates indicating that roughly 20–30% of HCV-infected patients experience depression—substantially above the prevalence in uninfected populations [26–28]. Analogous trends have been observed for HBV. In a clinical study involving 130 subjects, patients with chronic hepatitis B showed markedly higher depression scores on the Hospital Anxiety and Depression Scale (HADS)-Depression scores compared to healthy controls [29]. Likewise, an Iranian cohort study reported a depression prevalence of 19.8% among individuals with chronic HBV, compared to 11.3% in healthy participants [30]. Although large-scale studies remain limited, growing evidence supports an association between chronic viral hepatitis and depression. This nationally representative study offers a more robust evaluation.

The link between chronic viral hepatitis and depression is complex and not yet fully understood. However, multiple interrelated mechanisms have been proposed to explain this association. One of the key mechanisms involves immune-inflammatory pathways. Chronic hepatitis B and C infections induce a persistent inflammatory state characterized by elevated levels of pro-inflammatory cytokines, including interleukin-1β (IL-1β), interleukin-6 (IL-6), tumor necrosis factor-α (TNF-α), and interferon-γ (IFN-γ) [31, 32]. These cytokines can cross the blood-brain barrier (BBB) and enter the central nervous system [33]. Once in the brain, they activate macrophages/microglia, triggering neuroinflammation that subsequently leads to neuronal damage and functional impairments [34]. These cytokines disrupt neurotransmitter balance by altering the synthesis, release, or reuptake of monoamines, including serotonin (5-HT), dopamine (DA), and norepinephrine (NE) [35]. Since these neurotransmitters play crucial roles in perception, attention, mood regulation, and cognition, their dysregulation may contribute to the onset of depression [36]. Additionally, pro-inflammatory cytokines, particularly IL-6, have been implicated in dysregulation of the hypothalamic-pituitary-adrenal (HPA) axis, resulting in excessive cortisol production [37, 38]. This hyperactivation promotes hippocampal atrophy and reduces brain-derived neurotrophic factor (BDNF) levels, both of which are closely associated with the pathogenesis of depression [39]. Moreover, cytokines such as interferon-α (IFN-α) and IL-6 activate indoleamine 2,3-dioxygenase (IDO), an enzyme that shifts tryptophan metabolism toward the kynurenine pathway [40]. This metabolic shift reduces serotonin synthesis while increasing the production of neurotoxic metabolites, including quinolinic acid (QA) and kynurenic acid (KA), which further exacerbate neurotoxicity and contribute to depressive symptoms [41].

Beyond immune-inflammatory pathways, chronic hepatitis can influence mental health through other pathways. Although hepatitis viruses lack classical neurotropism like HIV, studies have detected genetic material and proteins in the brain [23, 42, 43]. This finding suggests that direct viral effects may play a role in the pathogenesis of depression. Additionally, living with chronic hepatitis can impose significant psychosocial stressors. Stigma and discrimination from family members, the workplace and even health-care workers foster shame and loneliness [44, 45]. Fear of disease progression and uncertainty about prognosis further intensify anxiety and depressive symptoms [45]. Many patients also face a financial burden from long-term monitoring, diagnostic testing, and antiviral treatment, which may contribute to psychological stress [46]. Finally, certain antiviral treatments, particularly interferon-based therapies for hepatitis C, have been shown to induce neuropsychiatric side effects, including fatigue and depression [47, 48]. Overall, these psychosocial and treatment-related factors not only heighten the risk of depression but can also lead to poor treatment adherence [49], diminished quality of life [50], and compromised disease management [51]. Therefore, addressing them should go beyond routine depression screening. Psychosocial support must be regarded as an important component of comprehensive hepatitis care.

This study exhibits several strengths. First, we used nationally representative NHANES data with appropriate sample weighting to ensure generalizability. Second, we applied rigorous statistical methods to enhance the robustness of our findings. However, this study has several limitations. First, the cross-sectional design precludes causal inference, and reverse causality cannot be excluded. Second, depressive symptoms were measured with a self-reported questionnaire, introducing potential recall and social-desirability bias. Third, although NHANES is nationally representative, it may underrepresent individuals with communicable chronic diseases such as HBV or HCV, which could affect the generalizability of the findings. Fourth, despite post hoc power analysis indicating adequate power, the small number of hepatitis virus–infected participants may yield imprecise estimates. Fifth, residual confounding may persist despite covariate adjustment. Finally, the exclusion of participants with missing covariate data may have introduced selection bias.

## Conclusion

Viral hepatitis was positively associated with depressive symptoms, and this relationship was confined to individuals with active infection. Patients with active HBV or HCV infection may be at higher risk of depression, highlighting the need for regular screening and support. Because the study is cross-sectional, causality cannot be inferred; longitudinal studies are required to confirm temporal and causal relationship.

## Supplementary Information

Supplementary Material 1. Model-fit statistics (AIC, BIC, Wald F) for all multivariable models.

Supplementary Material 2. Detailed methods, post-hoc power analysis and R code.

## Data Availability

There is public access to the data described in this study through the NHANES website: https://wwwn.cdc.gov/nchs/nhanes.

## Abbreviations

NHANES: National Health and Nutrition Examination Survey
PHQ-9: Patient Health Questionnaire-9
HBV: Hepatitis B Virus
HCV: Hepatitis C Virus
PIR: Poverty-Income Ratio
BMI: Body Mass Index
HADS: Hospital Anxiety and Depression Scale
IL-6: Interleukin-6
TNF-α: Tumor Necrosis Factor-Alpha
BDNF: Brain-Derived Neurotrophic Factor
IDO: Indoleamine 2,3-dioxygenase
QA: Quinolinic Acid
KA: Kynurenic Acid
CSF: Cerebrospinal Fluid
CI: Confidence Interval
OR: Odds Ratio
