# Supplementary material for: Association between viral hepatitis and depressive symptoms: National Health and Nutrition Examination Survey (NHANES) 2007–2018
Source: BMC Public Health. 2025 Sep 30;25:3161. doi: 10.1186/s12889-025-24346-z (PMC12487628; doi:10.1186/s12889-025-24346-z)
Supplement: Supplementary file 2 — Supplementary Material 2. Detailed methods, post-hoc power analysis and R code. [file 12889_2025_24346_MOESM2_ESM.docx]

Supplementary Material for: Association Between Viral Hepatitis and Depressive Symptoms

# 1. Post Hoc Power Analysis

Given that the prevalence of hepatitis B and C virus infection in the study population was only 1.7%, we performed a post hoc power analysis to assess whether the sample size was sufficient to detect the observed association between hepatitis virus infection and depressive symptoms (PHQ-9 ≥10).

Using the pwr package in R (version 4.4.3), we compared the proportion of depressive symptoms between infected (18.4%, n = 449) and uninfected (7.8%, n = 24,867) participants, as reported in Table 1 of the main manuscript. The calculated effect size (Cohen’s h) was 0.321. At a two-sided significance level (α = 0.05), the estimated power was 99.99991%, indicating that the current sample size was sufficient to detect the observed difference.

# 2. R Code

library(pwr)
 Define proportions
p1 <- 0.184 Proportion of infected participants with PHQ-9 ≥10
p2 <- 0.078 Proportion of uninfected participants with PHQ-9 ≥10

Calculate effect size h
h <- ES.h(p1, p2)
Define sample sizes
n1 <- 449 Number of infected participants
n2 <- 24867 Number of uninfected participants

 Conduct power analysis
power_result <- pwr.2p2n.test(h = h, n1 = n1, n2 = n2, sig.level = 0.05)

 Output results
print(power_result)

# 3. Output Summary

difference of proportion power calculation for binomial distribution (arcsine transformation)

h = 0.3205667
n1 = 449
n2 = 24867
sig.level = 0.05
power = 0.9999991
alternative = two.sided

NOTE: different sample sizes
